# On the biomechanical relationship between applied hip, knee and ankle joint moments and the internal knee compressive forces

**DOI:** 10.1080/23335432.2018.1499442

**Published:** 2018-09-13

**Authors:** Jonas Stensgaard Stoltze, John Rasmussen, Michael Skipper Andersen

**Affiliations:** Material and Production, Aalborg University, Aalborg, Denmark

**Keywords:** Knee joint forces, internal vs. external moment, inverse dynamics, in-silico, lower extremity, musculoskeletal modelling

## Abstract

Mechanical devices are common treating methods for knee osteoarthritis. It has the purpose of reducing the internal joint forces and unloading the damaged structure. The reduction is often achieved by alterations in the frontal plan, shifting the contact force from one compartment to the other, leaving the total compressive force unchanged. The aim of this study was to investigate how internal knee joint forces depend on applied external moments during gait. Musculoskeletal models of the gait of 10 healthy subjects were developed in the AnyBody Modelling System and used to simulate applied joint moments about different axes (load cases), each with the magnitude to compensate the net moment about the respective axis by a specified percentage. For each load case, the total, medial and lateral knee compressive force were computed and compared with a baseline case with no external moments applied. Among the investigated moments, hip flexion-extension, knee flexion-extension and ankle plantarflexion-dorsiflexion moment compensations have the most positive impact on the total knee joint compressive force, and combining the 3, each with a 40% compensation of the muscle moments, reduced the first peak by 23.6%, the second by 30.6% and the impulse by 28.6% with respect to no applied moments.

## Introduction

Osteoarthritis (OA) is a chronic, progressive, long-term and multifactorial joint disease with obesity, joint malalignment and joint laxity as some of the risk factors. The illness causes pain, stiffness and joint malalignment due to soft tissue deterioration in the affected joint (Amin et al. 2005), which limits mobility during activities of daily living and reduces quality-of-life (Silverwood et al. 2015). The number of OA patients has been growing and is expected to continue growing (Neogi 2015). In the absence of a cure (Hinman et al. 2012), there is a high demand for symptom management.

**Figure 8.**
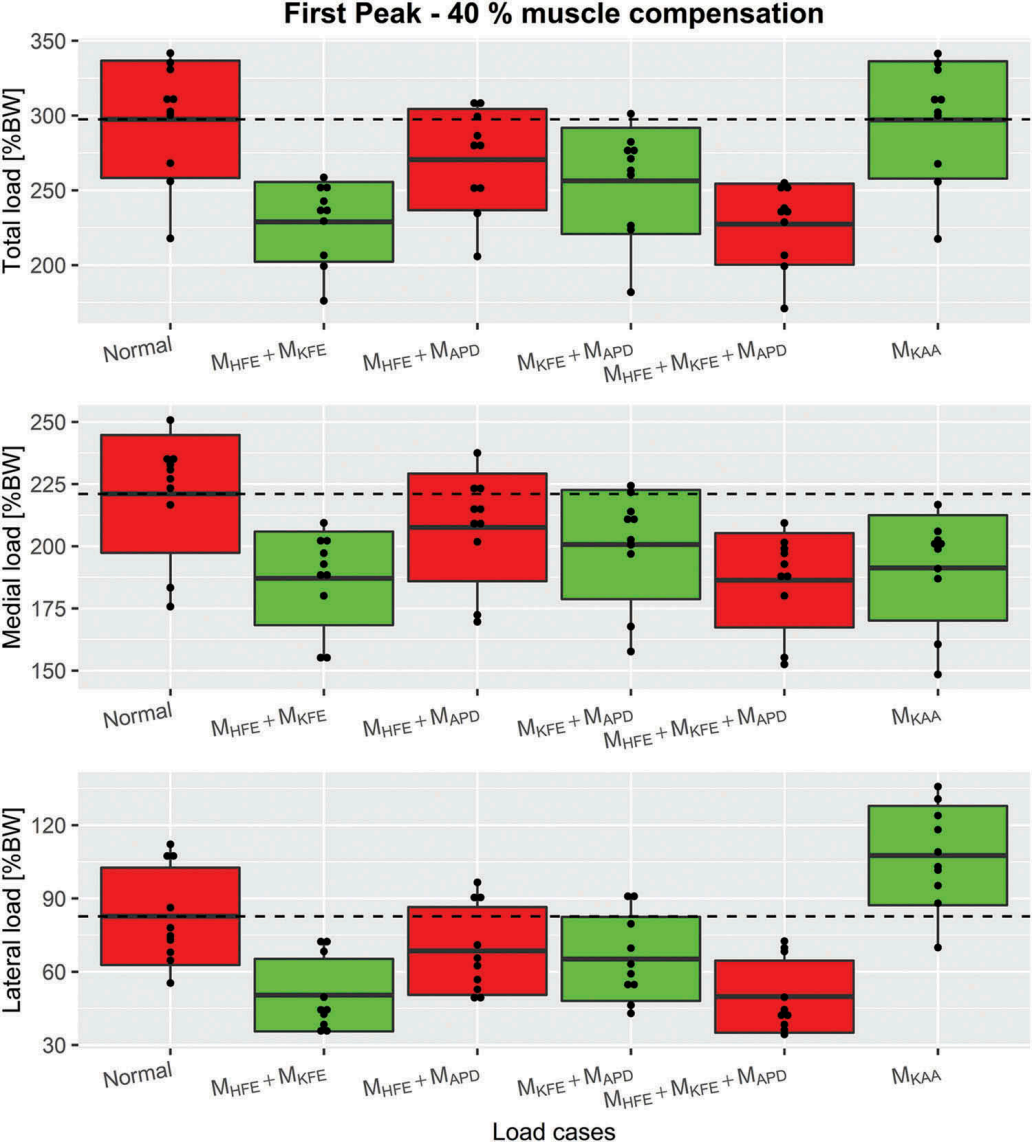
Boxplots, indicating the mean ± 1 standard deviation, of the first peak including Normal, M_KAA_ and combined load cases for each of the three compressive force types, top: the total compressive force, middle: medial force and bottom: lateral force. The dashed line represents the mean of Normal for visual comparison

**Figure 9.**
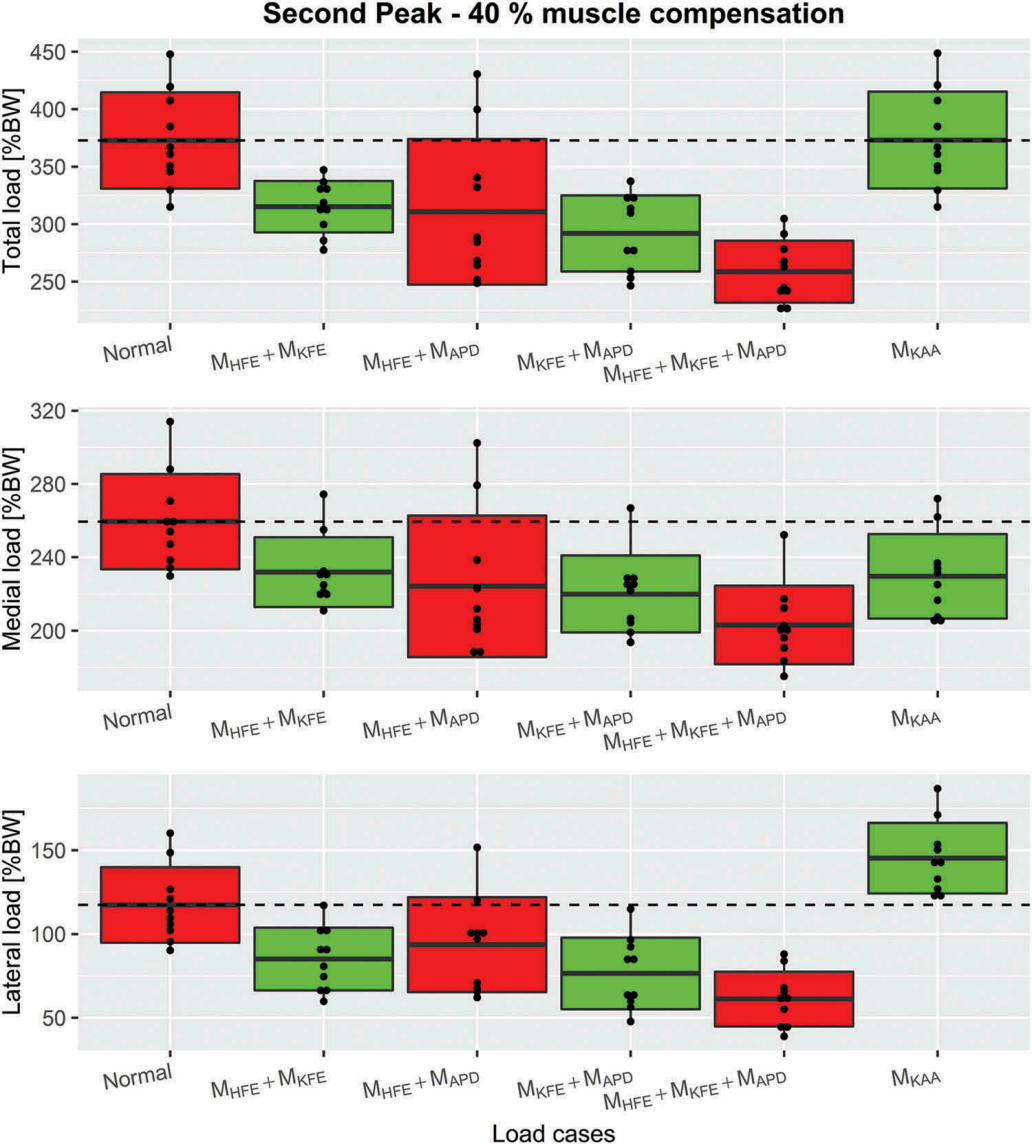
Boxplots, indicating the mean ± 1 standard deviation, of the second peak including Normal, M_KAA_ and combined load cases for each of the 3 compressive force types, top: the total compressive force, middle: medial force and bottom: lateral force. The dashed line represents the mean of Normal for visual comparison

Several intervention methods have been developed with the aim of reducing pain, increasing mobility and slowing down the progression of OA. These include physical therapy (Fitzgerald et al. 2016), shoe insoles (Skou et al. 2013), knee braces (Brooks 2014), ankle-foot orthoses (AFO) (Fantini Pagani et al. 2013) and surgery (Gardiner et al. 2016). This study deals with non-surgical treatment and focuses on the mechanical devices available for the lower extremity to reduce internal knee joint forces, since the knee is the most widely affected joint (Felson and Zhang 1998).

Internal joint forces are rarely measurable but crucial for treating OA since meniscus and cartilage deterioration rates depend on these contact forces and stress distribution among others (Johnson et al. 1980). Therefore, since OA often initiates in the medial compartment of the knee, the functionality of the main part of mechanical devices on the market is to shift the condyle force laterally to unaffected structures, often by reducing the external knee adduction moment (KAM), which can be achieved with both knee braces (Fantini Pagani et al. 2010) and AFOs (Fantini Pagani et al. 2013). However, the correlation between KAM and medial contact force has been debated in the literature. According to Walter et al. (2010), a reduced KAM does not guarantee a reduced internal medial compartment force during gait, since the joint compression forces from muscle contraction are not taken into account. Still, they found a good correlation for the second peak during stance phase whereas Kutzner et al. (2013) found the strongest correlation during early stance, which illustrates a non-conclusive relationship between the two variables. Furthermore, a correlation between KAM and cartilage damage was observed in a study by Brisson et al. (2016) but only for obese subjects with a body mass index over 30.

In general, interventions using mechanical devices have shown varying results, and scientific evidence of their biomechanical effect is still missing (Penny et al. 2013; Brooks 2014). One of the reasons might be that most devices, designed to relieve the contact forces in the knee joint, mainly focus on unloading one compartment and therefore applies moment in the frontal plane. However, this does not compensate for the contributions to the joint reaction forces caused by muscle contraction necessary to balance the joint moment in other planes. For both the knee (Jun et al. 2015) and the ankle (Collins et al. 2015), some devices have been designed to compensate moments in the sagittal plane, but it is not clear what externally applied moment reduces the internal knee joint load most efficiently. We present an investigation of the relationship between internal joint forces – both medial, lateral and total compressive forces – and external joint moments in both frontal and sagittal planes and taking into account active muscle forces. The purpose is to gain knowledge on how to reduce knee joint forces most efficiently, and since several of the muscles spanning the knee joint are bi-articular, interventions on the hip and ankle joints are assumed to affect the knee joint compressive forces as well. Therefore, we included interventions on the hip, knee and ankle by applying moments in-silico during gait while taking muscle contraction into account. To this end, we used musculoskeletal (MS) models developed in the AnyBody Modelling System (AMS) 6.0 (AnyBody Technology A/S, Aalborg, Denmark).

## Methods

### Computational methods

MS models developed in AMS from a previous study by Skals et al. (2017) of 10 healthy subjects (8 males and 2 females, age: 25.7 ± 1.5 years, height: 180.8 ± 7.4 cm, weight: 76.9 ± 10.4 kg), who performed, among others, 3 gait trials each, were applied with minor adjustments as will be explained later. The models were driven by full-body 3-D kinematics based on trajectories from 35 surface-mounted reflective markers (29 placed on the skin and 3 on each shoe) recorded with 8 infrared cameras (Oqus 300 series, Qualisys AB, Gothenburg, Sweden), sampled at 250 Hz. The ground reaction force (GRF) was sampled at 2000 Hz using two force plates (Advanced Mechanical Technology, Inc., Watertown, MA, USA). The study was performed in accordance with the regulations of the regional ethics committee.

The MS models were based on the GaitFullBody template from the AnyBody Managed Model Repository v. 1.6.3 and a detailed description of these can be found in the supplementary material.

Initially, AMS was used to perform inverse dynamic analysis of each gait trial for three different load cases in the sagittal plane: hip flexion/extension moment (M_HFE_), knee flexion/extension moment (M_KFE_) and ankle plantarflexion/dorsiflexion moment (M_APD_). For each load case, a parameter study was conducted in order to investigate how the reduction of internal knee joint force depends on the amount of the externally applied moment. This applied moment was specified to be between 0% and 100% of the moment generated by the muscles spanning the respective joint and incremented in steps of 20%. For example, when applying M_KFE_, this applied moment is equal to the specified percentage of the moment generated by the muscles responsible for creating the flexion/extension rotation about the knee joint, and thereby unloads the affected muscles in this particular direction throughout the entire gait cycle. These results were used to select the magnitude of the externally applied moment for the rest of the study, where the same type of inverse dynamics analysis was performed with the chosen compensation on four additionally load cases: Normal gait with no applied moments (Normal), applied hip abduction/adduction moment (M_HAA_), knee abduction/adduction moment (M_KAA_) and subtalar inversion/eversion moment (M_SIE_). The reason for only using one compensation percentage is based on the assumption of a close-to-linear trend between the amount of compensation and the amount of joint load reduction, and the amount was chosen as 40%, which is arbitrarily chosen since it depends on the application. All moments were applied in such a way that they either compensated for the muscles normally responsible for creating the movements (when applying M_HAA_, M_HFE_, M_KFE_, M_APD_ and M_SIE_) or counteracted the knee abduction/adduction (when applying M_KAA_) generated by the GRF, muscle contraction, inertia, gyroscopic and gravitational forces. After evaluating these seven load cases (Normal, M_HAA_, M_HFE_, M_KFE_, M_KAA_, M_APD_, M_SIE_,), combinations of the three with the largest reduction in the impulse during gait, based on MS analyses, were evaluated.

**Figure 1.**
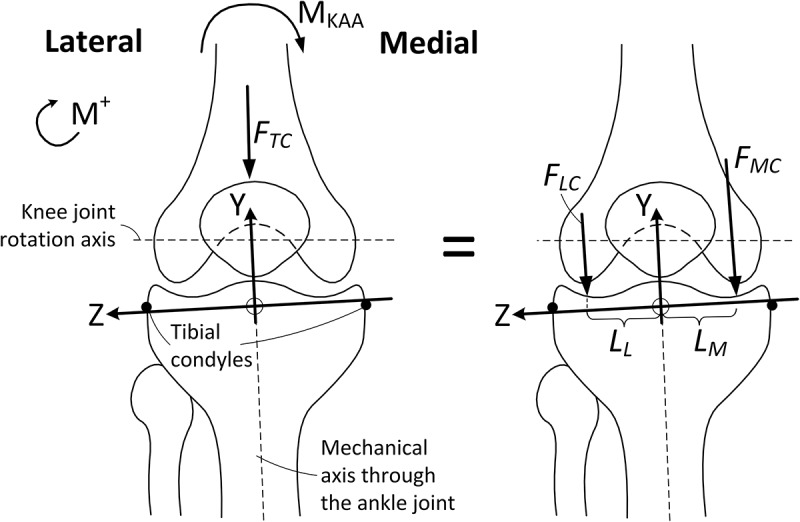
The tibial coordinate system in which all presented loads are defined. It is based on Grood and Suntay (1983) and a more detailed description can be found in the supplementary material. F_TC_ = total compressive force, F_MC_ = medial compressive force, F_LC_ = lateral compressive force, L_M_ = moment arm for the medial contact force, L_L_ = moment arm for the lateral contact force and M_KAA_ = the abduction/adduction moment about the *X*-axis, including contributions from external ground reaction loads, muscle forces, inertia forces, gyroscopic forces and gravity. M_KAA_ and F_TC_, given as F_TC_ = F_MC_ + F_LC_, are computed in AMS. M^+^ indicates the positive moment direction when formulating Equation (1)

For each load case, the total compressive knee joint force, F_TC_ and M_KAA_ were computed in AMS from which the medial and lateral compressive forces on the condyles, F_MC_ and F_LC_, respectively, were found in the tibial coordinate system by means of static equilibrium equations (1) in the frontal plane based on the free body diagram in Figure 1.

(1) $$F_{MC}=-\frac{F_{TC}L_{L}+M_{KAA}}{L_{L}+L_{M}}\;F_{LC}=-\frac{F_{TC}L_{M}-M_{KAA}}{L_{L}+L_{M}}$$

The medial and lateral moment arms, L_M_ and L_L_, respectively, were estimated from the relationships between internal knee geometry and the maximum width of the femoral condyles from medial to lateral sides as reported in Seedhom (1972).

### Data analysis

Each subject was represented by a mean contact force curve of the three gait trials normalized to bodyweight for each of the three contact force types (F_TC_, F_MC_ and F_LC_). These were used for further analysis to find the mean and the first and second peak values in the range of 10–20% gait cycle and 40–60% gait cycle, respectively, and also the impulse of each contact force type was found with numerical integration of the force curve by means of the trapezoidal method with unit spacing.

## Results

**Figure 2.**
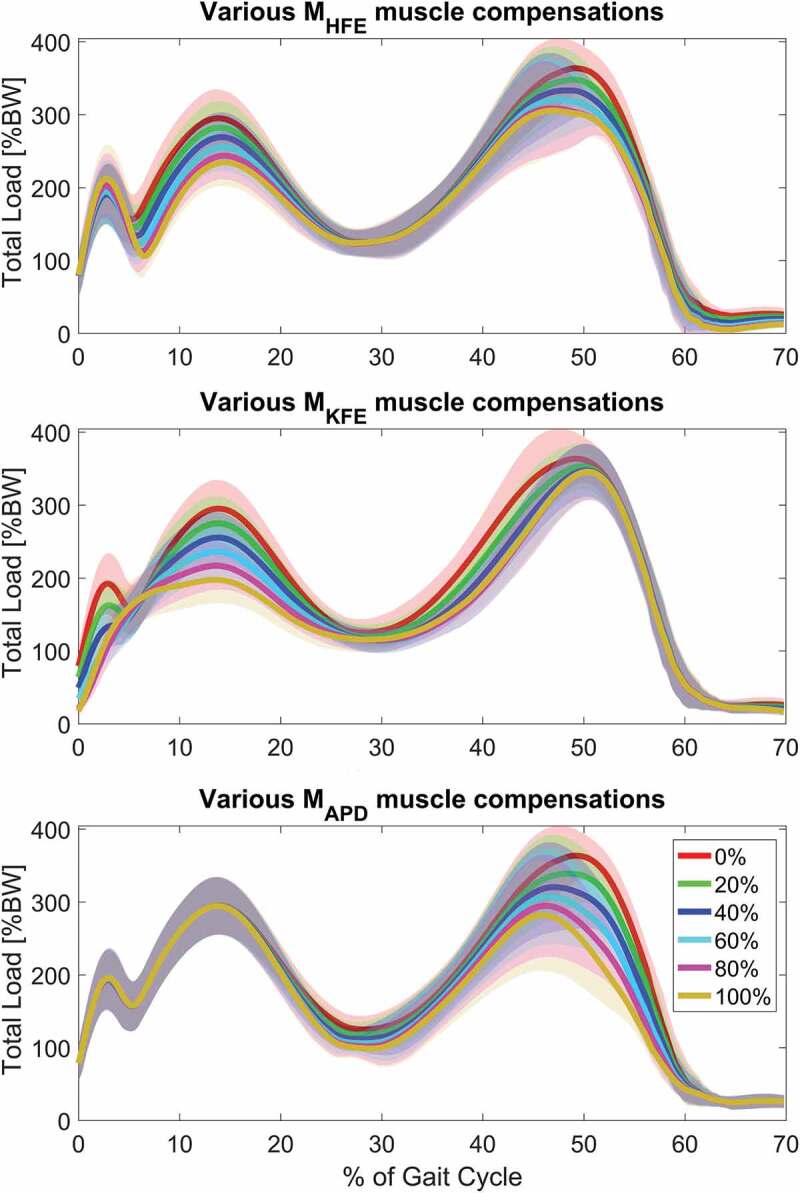
The total knee compressive joint load for muscle compensations of 0%, 20%, 40%, 60%, 80% and 100% of the three moments in the sagittal plane. The shaded area indicates ±1 standard deviation. According to the graphs, the potential for reducing internal joint loads, for applied moments in the sagittal plane, depends on the muscle activity

**Figure 3.**
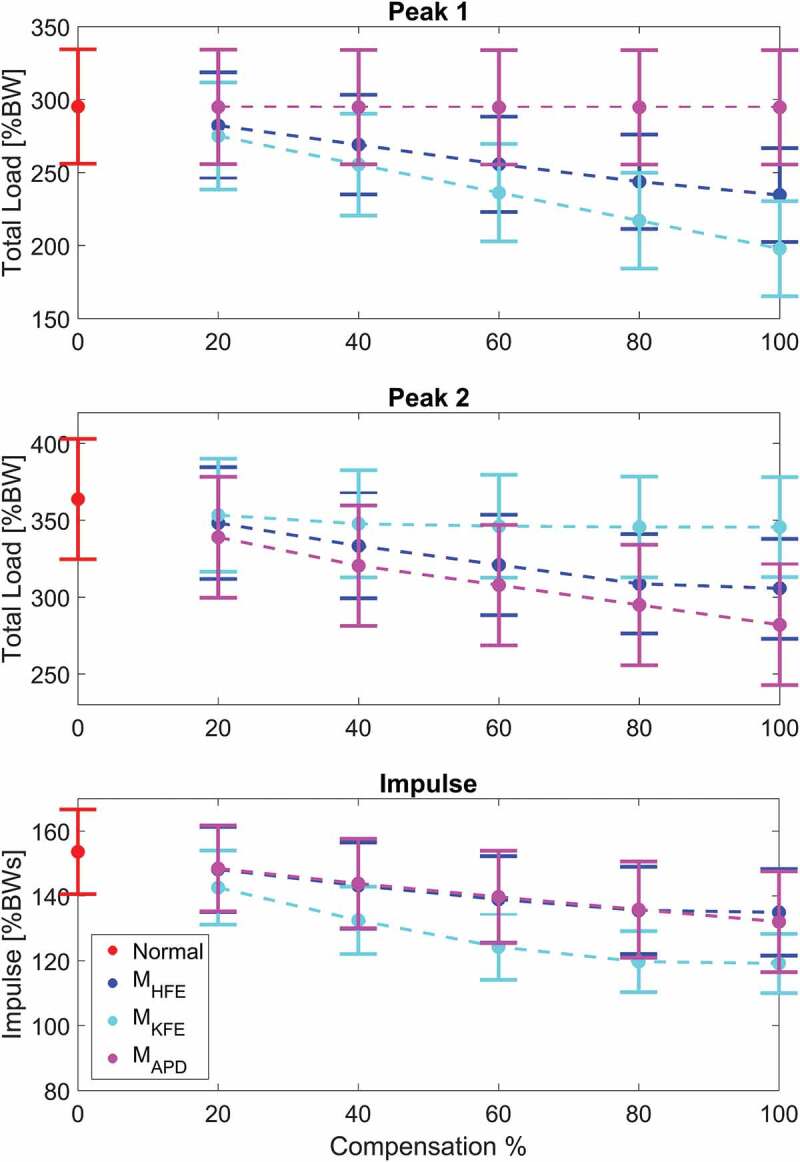
The mean peak values and impulse of the total load for the three moments in the sagittal plane as function of the amount of compensation. The whiskers indicates ±1 standard deviation and the dashed lines are visualising the trend between each simulated muscle compensation percentage. If any effect is present for the moment compensation, the relation is, according to the graphs, close to linear

Graphs on how the total compressive force, F_TC_, depends on the compensation percentage through the gait cycle are shown in Figure 2, and how the two peaks and impulse of the total compressive force is affected by compensation is illustrated in Figure 3.These two plots illustrate an almost linear relation between joint compression force and external moment compensation in the sagittal plane if the compensated muscles are activated during the investigated parameter. For example, when applying M_HFE_ or M_KFE_, the first peak decreases linearly with compensation percentage whereas M_APD_ does not influence the first peak. The same linear trend is present for the impulse where M_KFE_ has the biggest influence.

**Figure 4.**
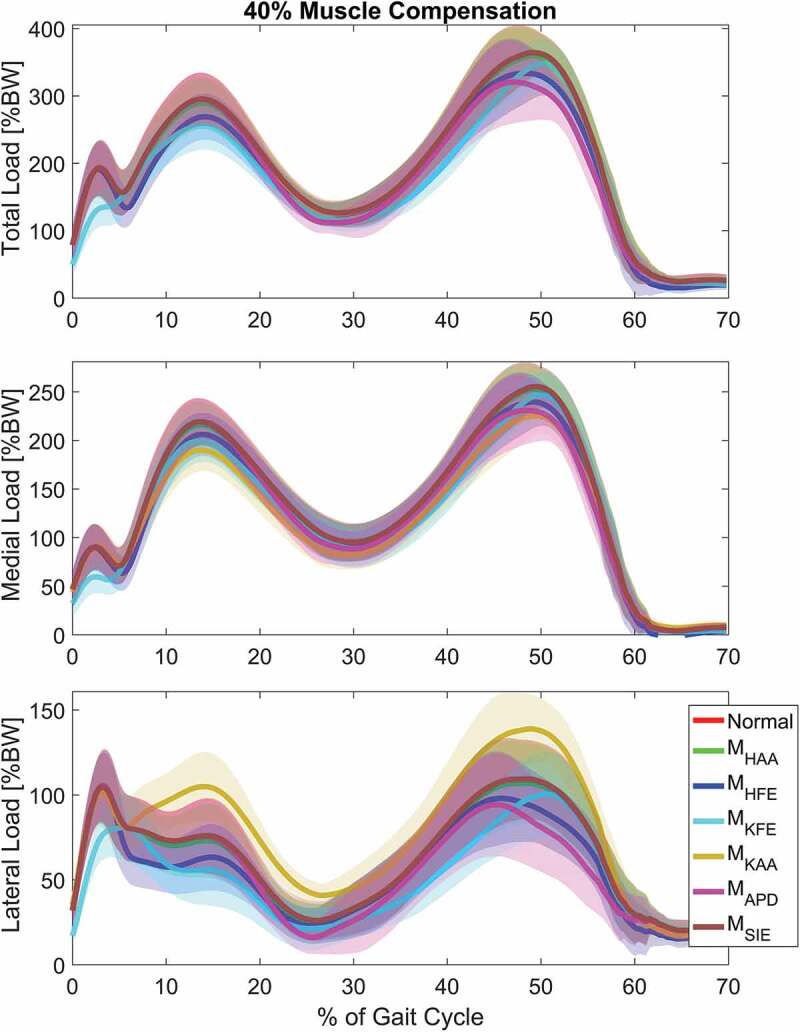
The mean internal knee joint load curves for normal gait when no external loads are applied (Normal) and single load cases applying 40% moment compensations for top: the total compressive force, middle: the medial condyle compressive force and bottom: the lateral condyle compressive force. The shaded area indicates ±1 standard deviation. The full gait cycle is from heel strike to heel strike but the swing phase has been omitted since the internal loads in this part are approaching zero for all load cases. As expected, the M_SIE_ and M_HAA_ have a very small influence in all three compressive force types for which reason they coincide with the red Normal line. Similarly, M_KAA_ also coincides with this line in the top figure since this applied moment only shifts the internal loads laterally leaving the total compressive load unaffected

**Figure 5.**
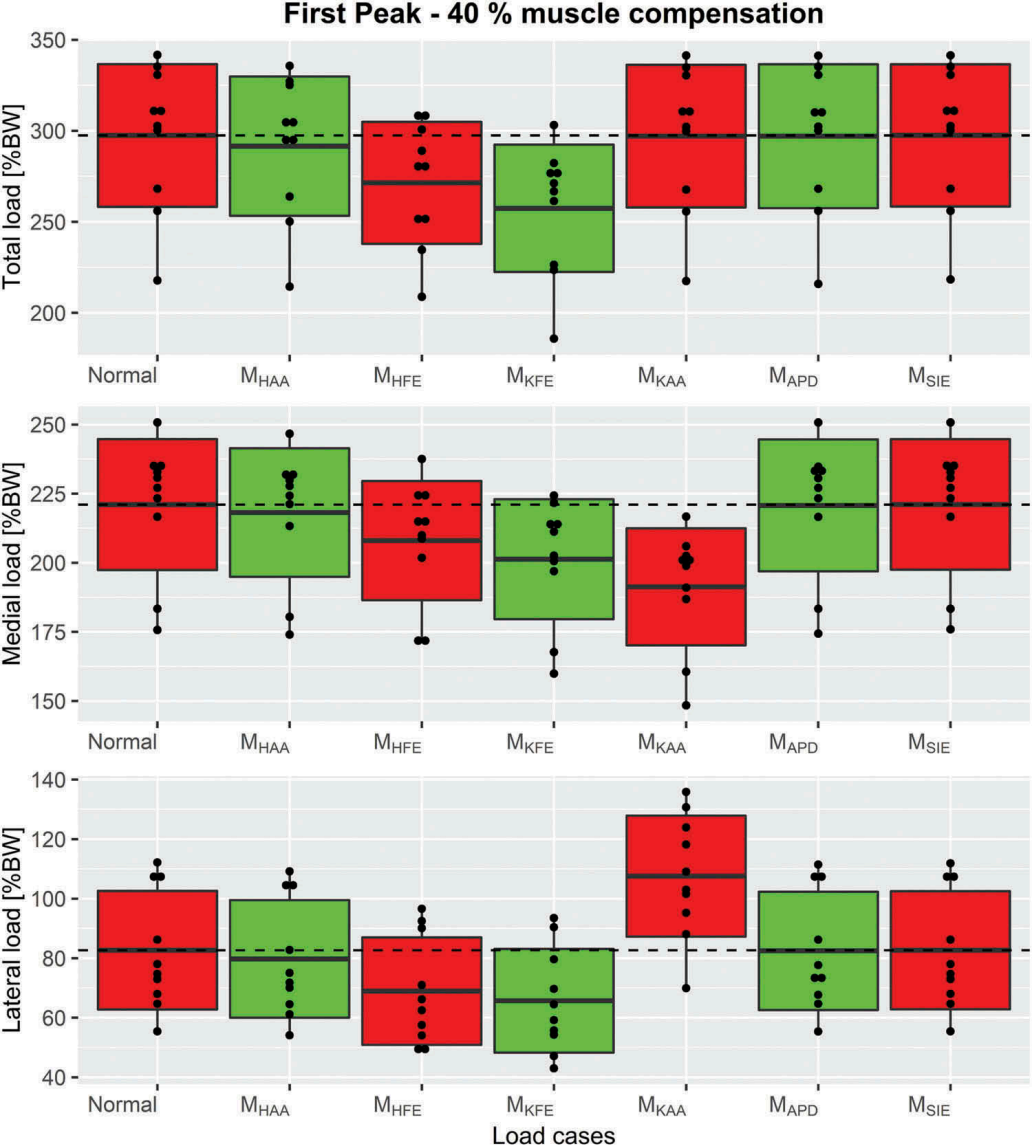
Boxplots, indicating the mean ± 1 standard deviation, of the first peak including Normal and the single load cases for each of the 3 compressive force types, top: the total compressive force, middle: medial force and bottom: lateral force. The dashed line represents the mean of Normal for visual comparison

**Figure 6.**
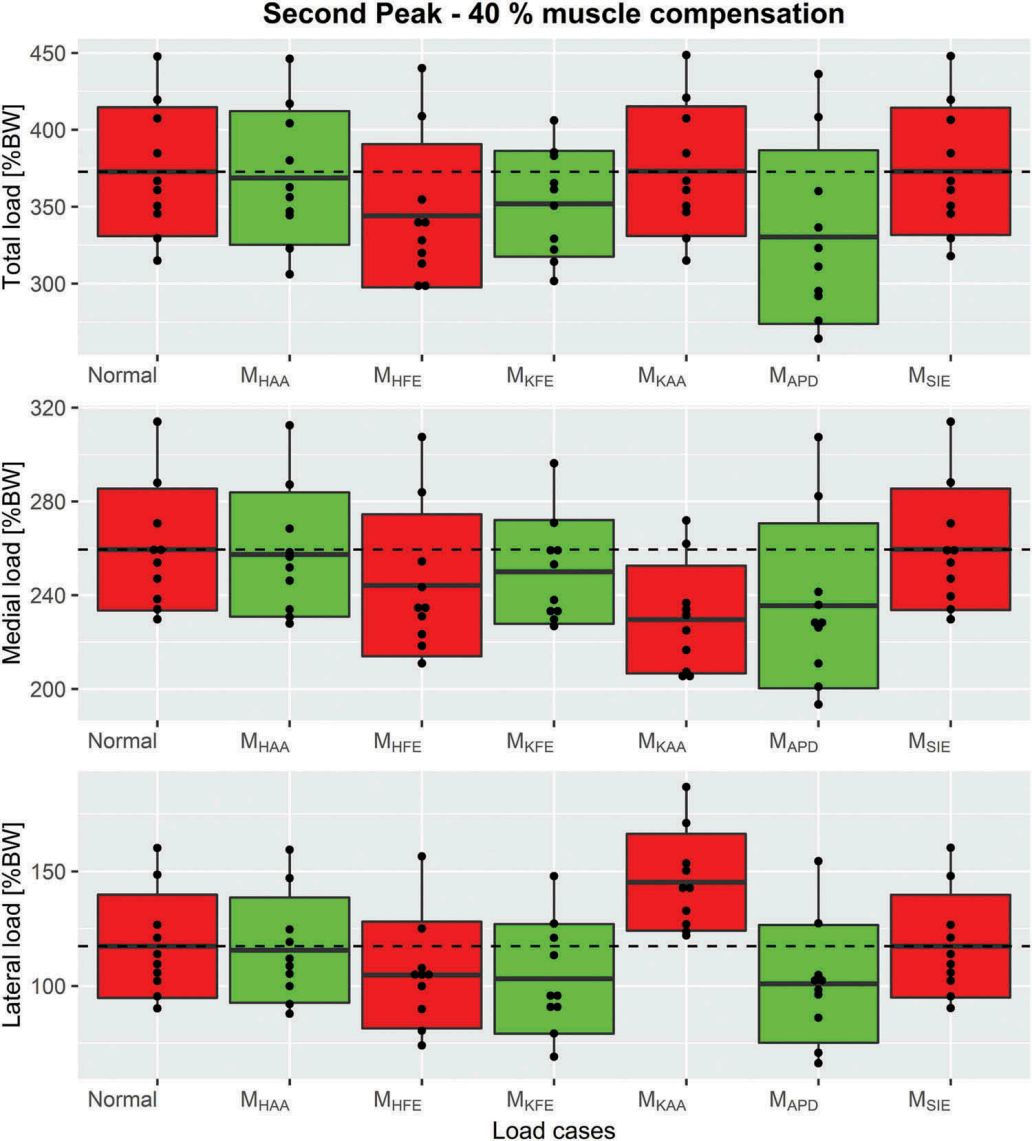
Boxplots, indicating the mean ± 1 standard deviation, of the second peak including Normal and the single load cases for each of the 3 compressive force types, top: the total compressive force, middle: medial force and bottom: lateral force. The dashed line represents the mean of Normal for visual comparison

The chosen compensation of 40% was applied for further analysis to compare the effect across the different load cases for each compression force: Total (F_TC_), medial (F_MC_) and lateral (F_LC_). Graphs on how these contact forces were affected through the gait cycle for each load case when applying single moment compensation of 40% are depicted in Figure 4 and illustrated with boxplots in Figures 5 and 6.

In general, the applied moments in the sagittal plane showed the largest effects. Both M_HFE_ and M_KFE_ significantly reduced the first peak mean of total compressive force relative to Normal by 8.8% and 13.5%, respectively (see Figures 4 and 5 top), whereas M_APD_ mainly affected the second peak by a reduction of 11.4%, which M_HFE_ reduced by 7.7% (see Figures 4 and 6 top).

Regarding condyle forces, M_KAA_ decreased the mean of first and second peak of medial force with 13.5% and 11.5%, respectively (see Figure 4–6 middle), but likewise increased the lateral force by 30.1% and 23.8% for the first and second peaks respectively (see Figures 4–6 bottom).

**Figure 7.**
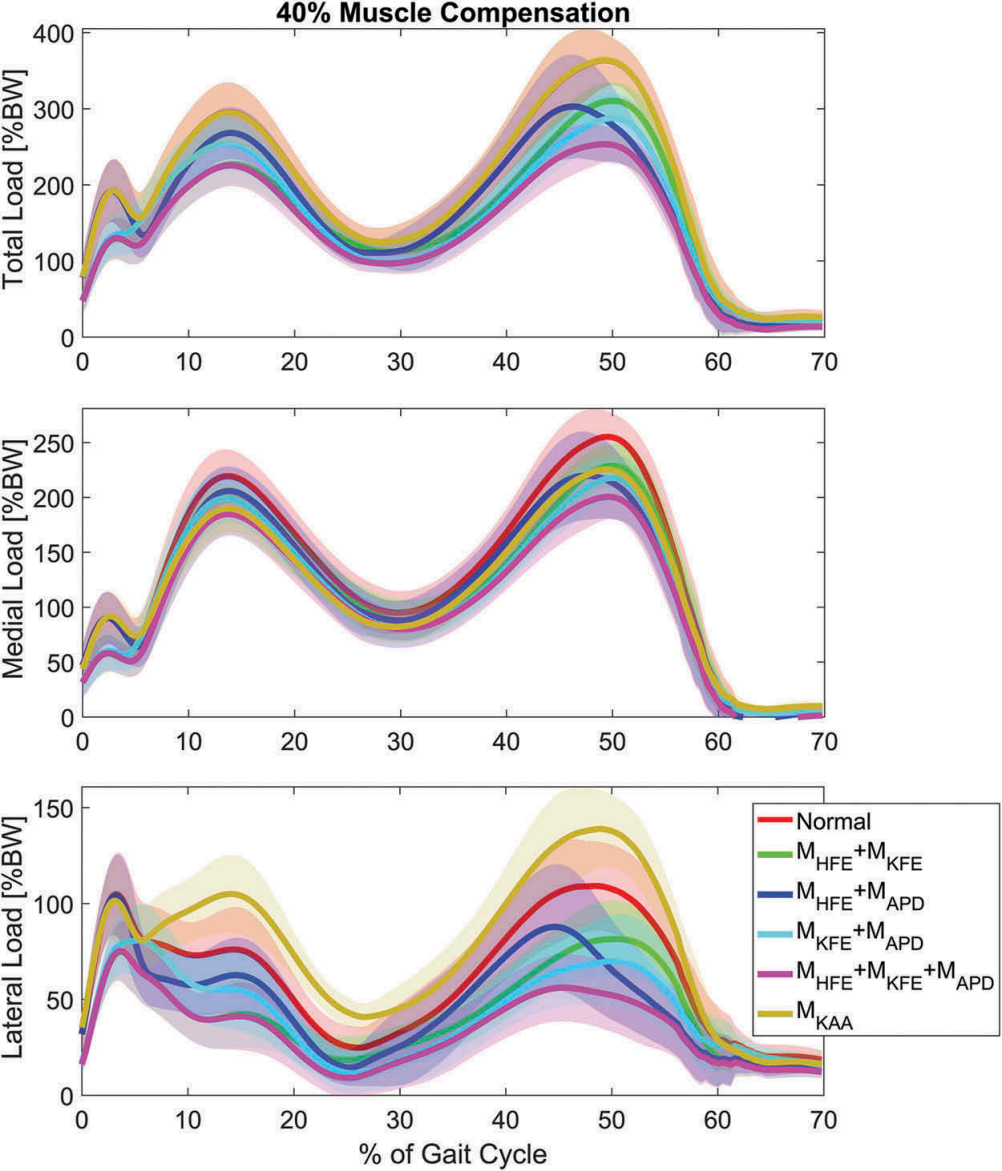
The mean internal knee joint load curves for normal gait when no external loads are applied (Normal) and combinations of applied 40% moment compensations for top: the total compressive force, middle: the medial condyle compressive force and bottom: the lateral condyle compressive force. The shaded area indicates ± 1 standard deviation. Similar to Figure 2, the swing phase has been omitted and the Normal load case is represented with the red lines for comparison

Plots of combined load cases are shown in Figures 7-9 which showed, that a combination of only M_HFE_ and M_KFE_ reduced the first peak mean of total compressive force with 23%, which is more or less the same as when including M_APD_. The second peak depends more on the number of combined moments. M_HFE_ + M_KFE_ and M_HFE_ + M_APD_ reduce the second peak by 15.5% and 16.7%, respectively, which is about half of the reduction when combining all three moments. However, M_KFE_ + M_APD_ increases the reduction to 21.7%. A major reduction was seen for medial, lateral and total compressive force when combining M_HFE_, M_KFE_ and M_APD_ which decreased the mean (over the trials) of the first peak (~13% gait cycle) and second peak (~50% gait cycle) for total compressive force by 23.6% and 30.6%, respectively, and the impulse was reduced by 28.6%.

Again, the M_HFE_ + M_KFE_ combination performed as well as M_HFE_ + M_KFE_ + M_APD_ on the first peak for both condyle forces: 15.4% and 39% mean reduction for medial and lateral condyle force, respectively. The second peak of the medial condyle force was reduced by 13.6% and 15.2% for M_HFE_ + M_APD_ and M_KFE_ + M_APD_, respectively, which a combination of all three moments increased to 21.7%. This combination reduced the second peak of the lateral condyle force by 47.9%, and reduced the impulse by 30.6%, 21.7% and 47.9% for total, medial and lateral force, respectively. However, the second best intervention regarding impulse was either M_HFE_ + M_KFE_ or M_KFE_ + M_APD_, which caused very similar reductions in all three investigated joint forces.

## Discussion

The purpose of this study was to investigate the relationship between externally applied joint moments and the internal joint forces at the knee, which provides information for the design of mechanical devices for unloading the knee joint in Knee OA (KOA) patients. When dealing with KOA, the medial contact force is mostly in focus since this is where the disease typically initiates; applying an external abduction moment seems like an obvious solution to shift the force laterally. Since this force distribution is not directly measurable, it is most often evaluated based on the external KAM, which, as reported by Walter et al. (2010), can be reduced without reducing the medial contact force. This can also be interpreted from Figure 4, which shows the second peak of the medial force to be the highest, mainly due to gastrocnemius muscle contraction (Schmitz et al. 2009), whereas the first KAM peak commonly is the highest during the weight acceptance phase.

Figures 2 and 3 show that for the three moments in the sagittal plane, the total knee compressive force reduction depends on the muscle activation; the more activation the larger force reduction is seen. For example, the APD load case only affects the second peak due to high gastrocnemius muscle activation, whereas this muscle has low activation during first peak (Schmitz et al. 2009) and hence no force reduction is observed at this state of the cycle for APD.

Our results indicate that an efficient approach regarding an overall reduction of medial, lateral and total compressive forces and impulse during gait, is applying a combination of hip and knee flexion/extension moment, knee flexion/extension and ankle plantarflexion/dorsiflexion moment, or a combination of all three. However, the practical application of combined hip flexion/extension, knee flexion/extension and/or ankle plantar-/dorsiflexion moments can be challenging since they each need to be active at different times during the gait cycle. As mentioned previously, the first load peak is purely affected by M_HFE_ and M_KFE_ since the hip and thigh muscles are mainly active here, but when approaching toe off and second load peak, the gastrocnemius muscle is activated more, for which reason the applied M_APD_ moment decreases the second peak more than M_KFE_ and M_HFE_. Based on this information, an orthosis can be developed which targets first peak by either compensating knee or hip flexion/extension moment and second peak by compensating either hip, knee or ankle joint moments in the sagittal plane of which the ankle, according to Figure 3, leads to the largest reduction. However, Wellsandt et al. (2016) concluded that decreased knee joint loading is associated with KOA for people who has suffered from anterior cruciate ligament injury, and reduced muscle strength is also considered as a risk factor for developing KOA (Thorstensson et al. 2004), which indicates that unloading of the knee joint should be done with care.

There are some limitations and uncertainties related to MS models and several parameters influences the joint loads (Moissenet et al. 2017). As shown in this study, the joint loads are highly affected by the surrounding muscles so the chosen muscle parameters have a big impact on the load reduction. Also, the muscle recruitment in AMS is based on an optimisation criterion (Damsgaard et al. 2006), which is an estimation of the real muscle configuration. When wearing a mechanical device applying external forces and/or moments to the lower extremity, the kinematics are most likely affected compared to an unbraced condition but since the moments in this study are applied in-silico, gait alteration is not taken into account. These changes most likely affect the joint forces since the contact with the ground, and thereby the muscle recruitment, changes. Lastly, the kinematics of the knee joint are highly complex during walking (Marra et al. 2015), which are not taken into account since the knee was modelled as an ideal hinge joint, in our simulations. However, despite these uncertainties the results clearly demonstrate, presuming ideal external moment application, a potential for substantial reduction in the internal knee joint forces while performing the same movement. Since the study only includes healthy subjects, it is uncertain if similar results are seen for KOA patients, which can be tested with similar analyses.

Since the knee internal–external (IE) muscle moment is relatively small compared to the other two knee moments, external IE moment was assumed to have only a minor effect on the compressive forces, and was therefore omitted. IE motion is often considered through foot progression angle since this changes the external KAM (Guo et al. 2007) by modifying the contact point between foot and the ground. IE motion in bracing has been introduced with the Odra brace (Orthoconcept Inc., Laval, QC, Canada), which additionally applies a distraction force to the knee during knee extension. To investigate the effect of IE motion, the knee joint in the MS model must be less constrained by the idealized joints and rather stabilized by the surrounding ligaments in the moveable directions. This is technically possible but will require a more advanced and computationally demanding model.

The results from this study indicate the contributions and ideal timing of external moments for reduction of internal knee compressive forces. Even though the highest reduction is seen for M_HFE_ + M_KFE_ + M_APD_ and combinations of two moments, these approaches seem technically challenging to realize with bracing. Thus, it might be necessary to limit the device to single moment compensation or two moments active separately, for example, M_HFE_ or M_KFE_ compensation for reducing first peak and M_APD_ for reducing second peak.

## Supplementary Material

Supplemental Material
