# Supplementary material for: On the biomechanical relationship between applied hip, knee and ankle joint moments and the internal knee compressive forces
Source: Int Biomech. 2018 Sep 13;5(1):63–74. doi: 10.1080/23335432.2018.1499442 (PMC7857478; doi:10.1080/23335432.2018.1499442)
Supplement: Supplemental Material [file TBBE_A_1499442_SM6352.docx]

Supplementary for the paper "On the biomechanical relationship between applied hip, knee and ankle joint moments and the internal knee compressive forces"

# Methods

## Experimental Procedure

The experimental study was conducted by Skals et al. (2017) at the Department of Health Science and Technology, Aalborg University, Denmark, where ten healthy subjects (8 males and 2 females, age: 25.70 ± 1.49 years, height: 180.80 ± 7.39 cm, weight: 76.88 ± 10.37 kg) volunteered to participate. During measurements, subjects wore tight fitting shorts, sports-brassiere for females and Brooks Ravenna 2 running shoes (Brooks Sports Inc., Seattle, WA, US) in preferred size.

Initially, a 5 min warm-up at 160 W was completed on a cycle ergometer before the gait cycle starting position was found through trial-and-error approach until the subjects were able to consistently impact the two force plates. Following, 29 markers were taped to their skin and three on each shoe; two representing the position of the first and fifth metatarsal and one maker at the top of the calcaneus bone. The reason for the excessive amount of markers is that the purpose of the experiment was to predict ground reaction forces and moments (GRF&Ms) for different activities, which requires full body kinematics. The marker locations are listed in Table 1 and illustrated in Figure 1, which indicates that no markers were placed on the head. Each subject completed five gait trials.

Table 1: The marker label, position and whether the marker positions were fixed (Fix.) or optimized (Opt.) in the anterior-posterior (A-P), medial-lateral (M-L) and proximal-distal (P-D) directions.

| **Label** | **Position** | **A-P** | **M-L** | **P-D** |
| --- | --- | --- | --- | --- |
| RTHI | Right thigh | Opt. | Opt. | Opt. |
| LTHI | Left thigh | Opt. | Opt. | Opt. |
| RKNE | Right lateral epicondyle | Fix. | Fix. | Fix. |
| LKNE | Left lateral epicondyle | Fix. | Fix. | Fix. |
| RPSI | Right posterior superior iliac spine | Fix. | Fix. | Fix. |
| LPSI | Left posterior superior iliac spine | Fix. | Fix. | Fix. |
| RASI | Right anterior superior iliac spine | Fix. | Fix. | Fix. |
| LASI | Left anterior superior iliac spine | Fix. | Fix. | Fix. |
| RANK | Right lateral malleolus | Fix. | Fix. | Fix. |
| LANK | Left lateral malleolus | Fix. | Fix. | Fix. |
| RHEE | Right calcaneus | Fix. | Fix. | Fix. |
| LHEE | Left calcaneus | Fix. | Fix. | Fix. |
| RTIB | Right tibia | Opt. | Opt. | Opt. |
| LTIB | Left tibia | Opt. | Opt. | Opt. |
| RTOE | Right metatarsus | Fix. | Fix. | Fix. |
| LTOE | Left metatarsus | Fix. | Fix. | Fix. |
| RMT5 | Right fifth metatarsal | Fix. | Fix. | Fix. |
| LMT5 | Left fifth metatarsal | Fix. | Fix. | Fix. |
| RELB | Right lateral epicondyle | Fix. | Fix. | Fix. |
| LELB | Left lateral epicondyle | Fix. | Fix. | Fix. |
| RWRA | Right wrist bar thumb side | Fix. | Fix. | Fix. |
| LWRA | Left wrist bar thumb side | Fix. | Fix. | Fix. |
| RFINL | Right first metacarpal | Fix. | Fix. | Fix. |
| LFINL | Left first metacarpal | Fix. | Fix. | Fix. |
| RFINM | Right fifth metacarpal | Fix. | Fix. | Fix. |
| LFINM | Left fifth metacarpal | Fix. | Fix. | Fix. |
| RUPA | Right triceps brachii | Opt. | Opt. | Opt. |
| LUPA | Left triceps brachii | Opt. | Opt. | Opt. |
| RSHO | Right Acromio-clavicular joint | Fix. | Fix. | Fix. |
| LSHO | Left Acromio-clavicular joint | Fix. | Fix. | Fix. |
| STRN | Xiphoid process of the sternum | Opt. | Opt. | Opt. |
| CLAV | Jugular Notch | Opt. | Opt. | Opt. |
| C7 | 7th Cervical Vertebrae | Fix. | Fix. | Fix. |
| RILC* | Right iliac crest | - | - | - |
| LILC* | Left iliac crest | - | - | - |

*Excluded

**Figure 1**: The marker placements used in Skals et al. (2017).

## Data Collection

The marker trajectories were recorded for the study in Skals et al. (2017) with eight infrared cameras (Oqus 300 series, Qualisys AB, Gothenburg, Sweden) sampling at 250 Hz and analyzed in Qualisys Track Manager v. 2.9. The laboratory had two force plates (width/length = 464/508 mm) (Advanced Mechanical Technology, Inc., Watertown, MA, US) embedded in the floor measuring the ground reaction force at 2000 Hz.

## Experimental Data Processing

A low-pass filter with second order, zero-phase Butterworth filters was used for the force plate and marker data using a cut-off frequency of 15 and 10 Hz respectively. The three of the five successful gait trials were included for further analysis, yielding a total of 30 trials used to investigate the effects of applied moments on internal joint loads. Trials were excluded due to occasional marker occlusion or inadequate impact of the force plates, i.e., the whole foot was not in complete contact or the impact occurred too close to the edges of the force plate surface.

## Musculoskeletal Model

The musculoskeletal (MS) models, used in this study, were developed by Skals et al. 2017 in the AnyBody Modeling System v. 6.0.4 (AMS) (AnyBody Technology A/S, Aalborg, Denmark) based on the GaitFullBody template from the AnyBody Managed Model Repository v. 1.6.3. The lower extremity model is based on the cadaver dataset of Klein Horsman et al. (2007), the lumbar spine model is based on the work of de Zee et al. (2007), and the shoulder and arm models are based on the work of the Delft Shoulder Group (Van der Helm et al. 1992, Veeger et al. 1991 and Veeger et al. 1997). The full MS model had a total of 29 degrees-of-freedom (DOFs); 2x1 DOFs for the ankle revolute joints, 2x1 DOFs for the subtalar revolute joints, 2x1 DOFs for the knee revolute joints, 2x3 DOFs for the hip spherical joints, 6 DOFs for pelvis, 3 DOFs for the rotation angles between pelvis and thorax controlled by a spine rhythm that distribute the angles between each vertebra, 2x1 DOF at the elbow revolute joints, and 2x3 DOFs at the glenohumeral spherical joints. Since no markers were placed on the head, the neck joint was fixed in a neutral position.

The kinematic analysis for all trials was solved based on the approach explained in Andersen et al. (2009) but prior to this, a linear scaling of the segments was applied on one gait trial for each subject with the method from Andersen et al. (2010). The scaling is based on the time varying model marker positions which are found with an optimization-based kinematic analysis with a weighted least-square objective function, tracking both the trajectory of the fixed markers on bony landmarks (Labeled Fix. in Table 1) and simultaneously the trajectory of the free-moving markers (labeled Opt. in Table 1). The calculated segment lengths and model marker trajectories were saved and used for the kinematic analysis for all other trials.

Regarding the inverse dynamic analysis, the lower extremity was actuated with a total of 110 muscles divided into 318 individual muscle paths. These were modelled as constant strength muscles whereas the muscles in the upper body were modelled as ideal joint torque generators. Muscle strength was scaled according to the mass-fat scaling explained in Rasmussen et al. (2005), and the muscle recruitment problem, computing the muscle and joint reaction forces, was solved by minimizing the sum of the squared muscle activities (Damsgaard 2006). This approach was validated with a tibial implant equipped with a six DOFs force measuring sensor in Marra et al. (2015) and showed promising results.

The internal knee loads, presented in this study, are found in a tibia coordinate system based on Grood and Suntay (1983) with origin half way between the two tibial condyles. The Y-axis (inferior-superior) is aligned with an axis from ankle joint to origin, Z-axis (medial-lateral) is perpendicular to this axis and goes through the right tibial condyle (when looking from posterior to anterior, so Z points laterally in the right leg and medially in the left leg). The X-axis (anterior-posterior) is the cross product of Y and Z and the rotation axis is based on Klein Horsman et al. (2007).

# References

Andersen, M. S., Damsgaard, M., Rasmussen, J., Andersen, M. S., Damsgaard, M., Rasmussen, J. 2009. Kinematic analysis of over-determinate biomechanical systems. Comput. Methods Biomech. Biomed. Engin. 12(4):371-384.

Andersen, M. S., Damsgaard, M., MacWilliams, B., Rasmussen, J. 2010. A computationally efficient optimisation-based method for parameter identification of kinematically determinate and over-determinate biomechanical systems. Comput. Methods Biomech. Biomed. Engin. 13(2):171-183.

Damsgaard, M., Rasmussen, J., Christensen, S. T., Surma, E., de Zee, M. 2006. Analysis of musculoskeletal systems in the AnyBody Modeling System. Comput. Methods Biomech. Biomed. Engin. 14(8):1100-1111.

de Zee, M., Hansen, L., Wong, C., Rasmussen, J., Simonsen, E. B. 2007. A generic detailed rigid-body lumbar spine model. J. Biomech. 40(6):1219-1227.

Grood, E. S., Suntay, W. J. 1983. A Joint Coordinate System for the Clinical Description of Three-Dimensional Motions Application to the Knee. J. Biomech. Eng. 105:136-143.

Klein Horsman, M. D., Koopman, H. F. J. M., van der Helm, F. C. T., Prosé, L. P., Veeger, H. E. J. 2007. Morphological muscle and joint parameters for musculoskeletal modelling of the lower extremity. Clin. Biomech. 22(2):239-247.

Marra, M. A., Vanheule, V., Fluit, R., Koopman, B. H. F. J. M., Rasmussen, J., Verdonschot, N., Andersen, M. S. 2015. A Subject-Specific Musculoskeletal Modeling Framework to Predict In Vivo Mechanics of Total Knee Arthroplasty. J. Biomech. Eng. 137(2):020904-1-020904-12.

Rasmussen, J., de Zee, M., Damsgaard, M., Christensen, S. T., Marek, C., Siebertz, K. 2005. A General Method for Scaling Musculo-Skeletal Models. International Symposium on Computer Simulation in Biomechanics, Cleveland, Ohio, United States

Skals, S., Jung, M., Damsgaard, M., Andersen, M. S. 2017. Prediction of ground reaction forces and moments during sports-related movements. Multibody Syst. Dyn. 39(3):175–195.
